# The Diagnostic Value of Mir-133a in ST Elevation and Non-ST Elevation Myocardial Infarction: A Meta-Analysis

**DOI:** 10.3390/cells9040793

**Published:** 2020-03-25

**Authors:** Yehuda Wexler, Udi Nussinovitch

**Affiliations:** 1 Rappaport Faculty of Medicine and Research Institute, Technion - Israel Institute of Technology, POB 9649, Haifa 3109601, Israel; yehuda.wexler@gmail.com; 2 Applicative Cardiovascular Research Center (ACRC) and Department of Cardiology, Meir Medical Center, Kfar Saba 44281, Israel

**Keywords:** myocardial infarction, MicroRNA, Mir-133, coronary heart disease, biomarker, meta-analysis

## Abstract

Numerous studies have reported correlations between plasma microRNA signatures and cardiovascular disease. MicroRNA-133a (Mir-133a) has been researched extensively for its diagnostic value in acute myocardial infarction (AMI). While initial results seemed promising, more recent studies cast doubt on the diagnostic utility of Mir-133a, calling its clinical prospects into question. Here, the diagnostic potential of Mir-133a was analyzed using data from multiple papers. Medline, Embase, and Web of Science were systematically searched for publications containing “Cardiovascular Disease”, “MicroRNA”, “Mir-133a” and their synonyms. Diagnostic performance was assessed using area under the summary receiver operator characteristic curve (AUC), while examining the impact of age, sex, final diagnosis, and time. Of the 753 identified publications, 9 were included in the quantitative analysis. The pooled AUC for Mir-133a was 0.73. Analyses performed separately on studies using healthy vs. symptomatic controls yielded pooled AUCs of 0.89 and 0.68, respectively. Age and sex were not found to significantly affect diagnostic performance. Our findings indicate that control characteristics and methodological inconsistencies are likely the causes of incongruent reports, and that Mir-133a may have limited use in distinguishing symptomatic patients from those suffering AMI. Lastly, we hypothesized that Mir-133a may find a new use as a risk stratification biomarker in patients with specific subsets of non-ST elevation myocardial infarction (NSTEMI).

## 1. Introduction

Cardiovascular disease (CVD) is the leading cause of death in the United States [1] and accounts for nearly $219 billion in spending annually and 647,000 deaths [2]. Coronary artery disease (CAD) is the most prevalent form of CVD with upwards of 365,000 American mortalities each year, primarily as a result of acute myocardial infarction (AMI) [2]. With over 800,000 Americans suffering AMIs annually [3], early detection is crucial to improving clinical outcomes and decreasing mortality.

Currently used circulating biomarkers such as cardiac troponins and creatine kinase MB act as sensitive and specific tests for myocardial damage, yet, they may be negative early in the process of ischemia. Their increase in the setting of ST-elevation MI (STEMI), a process that nearly always results from coronary plaque rupture and thrombosis formation, is usually reflective of the extent of the infarct and approximates the mass of cardiomyocytes that damaged in the process of AMI. In the setting of non-ST elevation myocardial infarction (NSTEMI), increases in different biomarkers may be suggestive of a specific underlying pathophysiology, although data is limited on such associations. For instance, it was suggested that extreme levels of cardiac troponins are suggestive of total occlusion (TO) of the culprit artery [4], but the results were not used to assess correlations with other entities of myocardial injury, and data on such a possible association is limited. At present, biomarkers are not used to differentiate specific coronary pathologies, or assess the extent of vascular occlusion, nor are they used to detect non-CAD related myocardial cell damage [5].

Early reperfusion, usually through percutaneous coronary intervention (PCI) is a primary factor in the prognosis and clinical outcome of AMI [6,7]. Although electrocardiographic signs of the ST segment elevation are a sensitive and specific sign of coronary TO in the setting of STEMI, approximately only 25.5–34% of NSTEMI patients were found to have TO [8,9]. Patients suffering from TO are commonly underdiagnosed, receive delayed intervention, and have increased rates of complications and mortality [8]. Additionally, it may be challenging to distinguish between NSTEMI resulting from coronary atherosclerosis, and other processes of myocardial damage associated with inflammation, microvascular damage, toxic injury, vasoconstriction, etc.

Thus, highly sensitive and specific circulating biomarkers capable of diagnosing AMI (and specifically patients with TO) shortly after symptoms begin are of great clinical importance and may reduce mortality as well as improve patient outcomes.

In recent years multiple circulating micro-RNAs (MiRNAs) have been identified and investigated for their possible diagnostic and prognostic utility in CVD [10,11,12,13,14]. Specifically, Micro-RNA 133a (Mir-133a) has been reported as a potentially powerful biomarker for AMI and CVD. Mir-133a is a short non-coding RNA molecule, which serves to regulate target genes through post-transcriptional suppression. It has been found that Mir-133a is critical for proper cardiac development, playing an important role in early differentiation and cardiogenesis, as well as mediating various cardiac processes including apoptosis, cardiac remodeling, hypertrophy, conductance, and automaticity [15]. Increased serum levels of Mir-133a have been observed in the setting of AMI and CVD. This is most likely the result of damaged myocardium releasing Mir-133a during cellular lysis, or adjacent border zone myocardium releasing Mir-133a containing vesicles in response to the cardiac insult [16]. The research on Mir-133a’s diagnostic potential, however, is strongly conflicted, with some papers reporting weak correlations between circulating Mir-133a concentrations and AMI [10,17], and others reporting strong correlations with excellent sensitivity and specificity [11,18,19,20,21,22]. In light of these contradictory findings, this meta-analysis synthesizes data from existing literature in order to examine the true potential of Mir-133a as a biomarker in AMI. Additionally, we analyzed the time frames in which Mir-133a was quantified, and their effect on the increases in plasma concentration, to ascertain whether Mir-133a may be useful as a very early diagnostic marker. Lastly, we compared the data of STEMI patients with those of NSTEMI patients to determine if Mir-133a might be used to distinguish between these two types of AMI. We hypothesized that TO in the setting of NSTEMI, and other specific entities of myocardial injury, may be characterized by distinct Mir-133a increase patterns, and aimed to evaluate the literature in that regard. Notably, correct identification of high risk NSTEMIs has a critical impact on the course of treatment [8], and therefore Mir-133a may serve as a valuable biomarker in this respect.

## 2. Materials and Methods

### 2.1. Search Strategy

Three electronic databases (Pubmed, Embase, and Web of Science) were searched for articles written prior to December 1st, 2019 that included the terms microRNA, microRNA-133, and cardiovascular disease, as well as common synonyms for these terms. The complete search strategy for all databases can be found in the Supplementary Materials (Tables S1–S3) in accordance with PRISMA guidelines [23].

### 2.2. Inclusion and Exclusion Criteria

All papers retrieved in the literature search were subjected to the following criteria for inclusion:

1. STEMI or NSTEMI was the clinical diagnosis in study patients.
2. The study was either case-controlled or a cohort.
3. Mir-133a was quantified from plasma using qRT-PCR with either SYBR or TaqMan probes.
4. Sample size, area under the standard receiver operator characteristic curve (AUC), location of study, and maximum plasma sample collection time must be stated.
5. A sample size of 5 or more patients was required for each subgroup.

The following criteria were used for exclusion:

1. Papers written in languages other than English.
2. Reviews, meta-analyses, posters, and correspondence letters.
3. Experimental design based solely on animal models.

Studies meeting all the inclusion criteria and none of the exclusion criteria were used for the quantitative analysis. If two or more included papers were based on the same clinical data, only the most relevant study was included. Screening was performed in accordance with PRISMA guidelines [23]. The completed checklist can be found in the Supplementary Material (Table S4).

### 2.3. Data Extraction

All data used in this meta-analysis were extracted from the published versions of the included papers, their supplementary materials, and their referenced sources. No unpublished data was acquired for this analysis.

In papers where increases of Mir-133a were presented as a change in cycle threshold (ΔCT) compared to a predetermined reference, or as ΔΔCT (ΔCT test sample–ΔCT calibrator sample), fold change was calculated using 2^(−ΔCT) or 2^(−ΔΔCT). Additionally, when fold changes were presented only for subgroups (for example STEMI and NSTEMI), the total fold change was calculated using a weighted average based on the number of patients in each subgroup.

When the demographic characteristics of subgroups were not specified in the papers (i.e., mean age, gender, etc.), it was assumed that they followed the same distribution as the larger group whose characteristics were listed.

In several papers [10,24] quantitative data was presented graphically without exact numbers being published. In these cases, the graphs and figures were digitized using GetData Graph Digitizer software Ver. 2.26 in a blinded manner, and the averaged numeric values were used in this analysis.

### 2.4. Statistical Analysis

The Kruskal–Wallis H test was used to compare findings between the groups. Relations between the dependent variables (fold change and AUC) and independent variables (percentage of STEMI patients (%STEMI), time from onset, and patients’ age) were evaluated using linear regression analysis. Correlation analyses were estimated according to the strength and direction of a linear relationship between the two variables on a scatterplot (i.e., r). The number of patients was used as a frequency weighted variable. A *p*-value of <0.01 was considered statistically significant. Pooled results were expressed as mean and standard deviation for AUC and as mean and standard error of mean (SEM) for fold changes. Analyses were performed using JMP version 7.0 (SAS Institute, Cary, NC, USA) and MedCalc version 19.1.5 (MedCalc Software Ltd, Belgium). Forest plots were generated using DistillerSR Forest Plot Generator (Evidence Partners, Ottawa, ON, Canada).

## 3. Results

### 3.1. Literature Search Results

**Figure 1:**
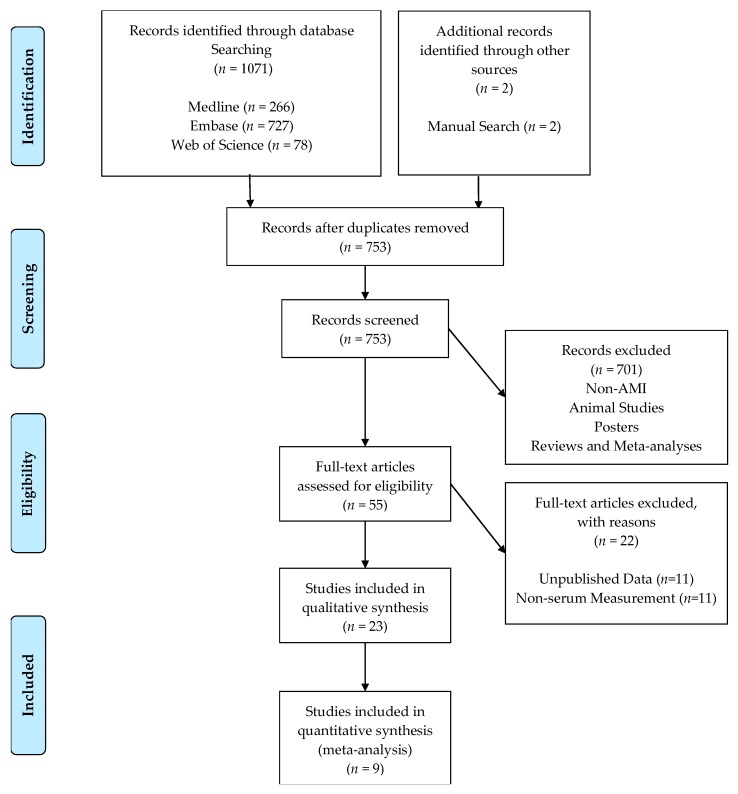
PRISMA flow chart of the study selection.

The literature selection process is shown in Figure 1. In short, the search returned 1071 results. After removal of duplicates 753 papers remained that were then assessed manually using titles and abstracts for: reviews, posters, meta-analyses, animal studies, and non-AMI related papers, which were all excluded.

We further screened the remaining 55 papers for relevance using their full texts, excluding all papers not written in English, papers, which did not publish their statistical data, and studies in which non-plasma samples were used for the quantification of Mir-133a.

The remaining 23 eligible studies [10,11,12,16,17,18,19,20,21,22,24,25,26,27,28,29,30,31,32,33,34,35,36] were thoroughly analyzed and subjected to the above inclusion criteria ultimately yielding 9 studies (Table 1) involving 2280 participants, with 943 AMI patients and 1337 controls that were included in the quantitative meta-analysis.

### 3.2. Meta-Analysis Results

**Figure 2:**
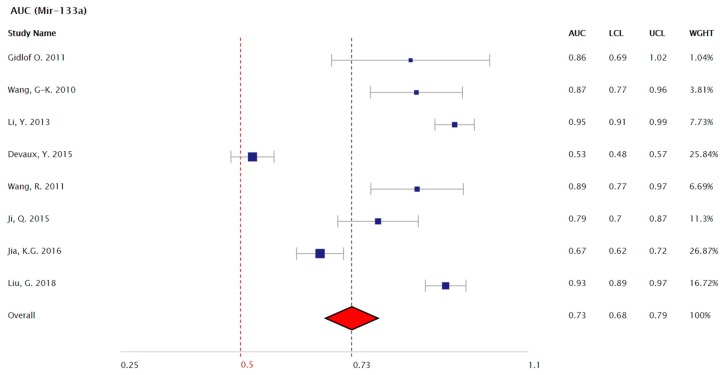
Forest plot of Mir-133a AUC values for the detection of AMI for each of the included studies. Pooled AUC value of 0.73 (95% CI 0.68–0.79). It is important to note that 5 out of 8 studies yielded AUC values greater than 0.86, yet, their overall weight was reduced by the relatively low number of included subjects.

The most consistently reported value in all the included studies was AUC. The combined frequency weighted analysis of this parameter yielded a pooled AUC of 0.73 (95% CI 0.68–0.79) for the eight studies [10,11,17,19,20,21,22,24] in which a 95% confidence interval of the AUC was provided (Figure 2).

**Figure 3:**
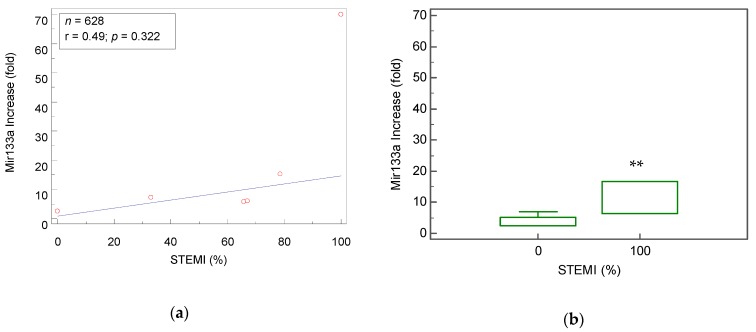
Linear regression analysis of (**a**) a relative increase (in fold) of Mir-133a plotted as a function of the percentage of patients in study with ST elevation myocardial infarction in composite groups. (**b**) Boxplot comparison of fold change in subgroups of 100% STEMI patients vs. 0% STEMI. ** *p* < 0.001.

Further subgroup analysis was performed in order to determine whether the distinction between STEMI and NSTEMI might account for some of the conflicting reports regarding increased Mir-133a concentrations following AMI. Relying on six papers that reported the percentage of STEMI patients in the study [17,18,20,21,24,37], and a linear regression model plotting Mir-133a fold as a function of this percentage, we found a moderate correlation (*r* = 0.49), with a trend of increasing Mir-133a concentration with higher percentages of STEMI patients (Figure 3a).

Furthermore, we compared subgroups from studies that reported data separately for STEMI or NSTEMI patients [17,18,20,21,24] and found a significantly higher value (*p* < 0.001) of the Mir-133a fold increase in STEMI patients vs. NSTEMI patients (11.6 ± 0.72 fold vs. 4.5 ± 0.14 fold, respectively; Figure 3b). Unfortunately, nearly all of the studies did not provide AUC data for these subgroups, and as such this parameter could not be analyzed. 

**Figure 4:**
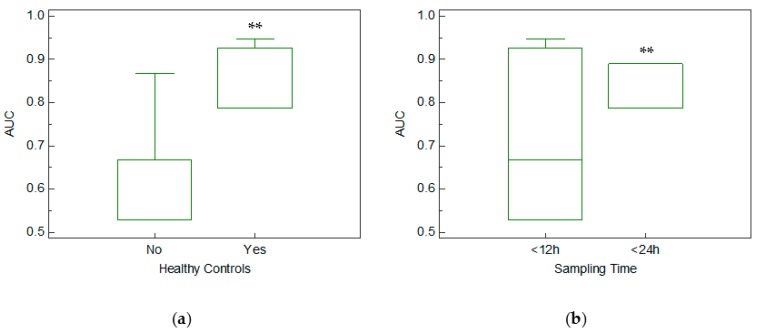
(**a**) Boxplot comparing AUC values based on control group characteristics (AUC was 0.89 ± 0.06 and 0.68 ± 0.14 when healthy or unhealthy controls were used, respectively), * *p* < 0.001. (**b**) Boxplot comparing AUC values based on sampling time (AUC was 0.82 ± 0.05 and 0.71 ± 0.01 for studies in which samples were acquired within 24 and 12 h, respectively). ** *p* < 0.001.

Of the included studies, four [10,11,18,22] were controlled with non-AMI patients presenting with symptoms of acute coronary syndrome (ACS), four used healthy volunteers [19,20,21,24], and one used a mixed control population, with a majority of non-healthy recruits [17]. In order to assess whether choice of control partially explains the discrepancies in reported AUC values for Mir-133a in AMI, we compared these two groups using boxplots (Figure 4a). We found that the AUC was significantly greater (*p* < 0.001) in studies that recruited healthy controls as opposed to those who used non-healthy controls (pooled AUC of 0.89 ± 0.07 vs. 0.68 ± 0.14, respectively).

In the included studies there was diversity in the percentage of male participants and mean age. To examine whether these factors impacted upon the reported results we used a linear regression model and found that both age and gender had little effect upon the reported results (Supplementary Figures S1 and S2).

Lastly, we divided the studies into two groups based on the reported time from onset of symptoms until sample acquisition. Of the included studies, six were conducted such that all samples were acquired within 12 h [10,17,19,20,21,22], two reported sample acquisition within 24 h [11,24], and one did not list a maximal time for sample acquisition and, as such, was not included in this analysis [18]. A significantly higher AUC value (*p* < 0.001) was found in the 24 h group when compared to the 12 h group (pooled AUC of 0.825 ± 0.05 vs. 0.715 ± 0.16 respectively; Figure 4b).

## 4. Discussion

AMI is a common and often deadly medical emergency. Presently, there is a great need for a quick and accurate diagnosis of AMIs, as well as improved methods for the detection of high-risk patients such as those with TO of the culprit artery, not presenting with STEMI. In the present study the diagnostic value of Mir-133a was analyzed to determine whether it may serve as a biomarker for very early detection of AMI, and to evaluate the contested claim that it may be useful in distinguishing STEMI from NSTEMI.

### 4.1. Mir-133a As an Early Biomarker for the Diagnosis of AMI

Historically, commonly used biomarkers for the diagnosis of acute myocardial infarction, such as cardiac troponins and creatine kinase MB, were not effective at very early diagnosis of AMI (within 0–3 h) [5]. Today, with the advent of high-sensitivity cardiac troponin tests, AMI can be diagnosed with reasonable accuracy even within the first hour [38,39]. Nevertheless, the search for additional early biomarkers, especially ones with different underlying molecular mechanisms, may allow for even greater sensitivity and specificity in shorter time frames.

To this end, early studies on Mir-133a reported high (>0.86, and as high as 0.95) AUC values [11,18,19,21,22], while some of the subsequent studies found lower sensitivities and specificities [10,17,24]. In this meta-analysis it was found that the pooled AUC for Mir-133a was 0.73 (95% CI 0.68–0.79). This value highlights its relatively weak sensitivity and specificity for the diagnosis of AMI, especially when measured against current troponin based methods that have markedly greater sensitivities and specificities [38,39]. Furthermore, it has yet to be shown whether Mir-133a, even in combination with current biomarkers, offers any diagnostic advantage and as such, its clinical value for the early diagnosis of AMI remains unclear. Notably, only two of the included papers suggested threshold values for the optimal diagnosis of AMI [17,18], highlighting the current lack of standardized values and measurements.

Additionally, we attempted to establish whether Mir-133a might be a more effective biomarker in the earliest stages of AMI, as was reported by Ji et al., 2015 [24]. Unfortunately, it is difficult in practice to determine how long after the onset of symptoms the samples are taken, and most studies did not report precise time frames. Therefore, we were only able to subdivide the studies into two main groups: (1) measurements made within 12 h [10,17,19,20,21,22] and (2) measurements made within 24 h [11,24]. The results of this analysis showed that, contrary to prior studies [11,24], measurements made within 24 h had a significantly higher pooled AUC. Regrettably, this does not settle the issue, as the distribution of individual measurements within each study is unclear, and only two studies were included in the 24 h group. Yet, the fact that measurements conducted within 12 h from symptoms yielded AUC values as high as 0.95 according to some reports [19], might be suggestive of Mir-133a’s potential as a diagnostic tool in specific patient populations. Further research, with larger sample sizes, as well as careful and repeated time measurements, are necessary in order to clarify the plasma concentration dynamics of Mir-133a in various conditions associated with myocardial damage.

### 4.2. Mir-133a in Healthy and Unhealthy Controls

A significant methodological issue regarding research on the diagnostic potential of Mir-133a is the choice of controls. As mentioned above, choices included healthy volunteers, patients with comorbidities and acute chest pain, and mixed populations. We suspected that the reported AUCs might have been greatly affected by the choice of controls, thus further obfuscating the clinical potential of Mir-133a. After analyzing the studies separately, based on the controls used, we found, in agreement with Jia et al. 2016 [17], that Mir-133a had a significantly higher (*p* < 0.001) pooled AUC of 0.89 ± 0.06 when healthy controls were used in comparison to 0.68 ± 0.14 in unhealthy. Hence, it may be concluded that Mir-133a can more efficiently distinguish between AMI patients and healthy volunteers, but it is not nearly as effective when testing patients presenting with symptoms of AMI. This may partially explain the apparent discordance in the existing literature. As increases in Mir-133a concentration are indicative of myocardial damage [30], it is reasonable to assume that a larger overlap will exist between patients presenting with acute chest pain and AMI patients than between healthy volunteers and AMI patients. For this reason, it may be concluded that Mir-133a might have greater diagnostic potential in patients presenting without classic symptoms of cardiac distress. If true, it can be speculated that Mir-133a might be of clinical importance in detecting troponin-based false-diagnosis of AMI in certain populations [40]. Current medical literature contains little information on this topic, and this speculation needs to be further evaluated in prospective studies.

### 4.3. Mir-133a As a Biomarker that Distinguishes Between STEMI and NSTEMI

In addition to early diagnosis of AMI, a clinical need for the detection of NSTEMI with TO of the culprit artery, as well as other serious cardiac conditions associated with a lack of ST segment elevation, exists. Due to inherent limitations in the standard 12-lead ECG, a complete electrocardiographic picture of the heart is not obtained. As a result, patients with acute occlusion of a coronary vessel may present with NSTEMI, and, as studies have shown [8], patients suffering from complete culprit artery occlusion presenting with AMI and no ST segment elevation are at a higher risk for mortality and adverse cardiac events than their ST elevated counterparts. It is believed that this is primarily due to the delay in identification and, as a result, establishment of reperfusion. Although several factors have been suggested to aid in the identification of patients with TO (such as prolonged duration of continuous chest pain, higher levels of the creatine kinase-MB fraction [41], and higher levels of high sensitivity troponin [4]), currently a delay of more than 24 h before an invasive procedure is performed, is common according to previous reports [8]. Furthermore, although patients with TO were reported to have higher mean Global Registry of Acute Coronary Events (GRACE) scores compared to those suffering subtotal occlusion (STO; 131 (range of 120–140) vs. 117 (range of 104–126); *p* = 0.032) [4], these values are still lower than the proposed cutoff of 140, which serves as an indication for the performance of PCI within 24 h according to accepted practice [8].

Consequently, it is of great importance to develop new and improved risk stratification tools that will allow clinicians to recognize high risk NSTEMI patients, and specifically those with occult TO, as early as possible.

Our results show that there was a trend of increasing Mir-133a plasma concentrations as the percentage of STEMI patients in the study grew. Moreover, we found a significantly greater increase (*p* < 0.001) in Mir-133a concentration amongst subgroups containing only STEMI patients vs. only NSTEMI patients. These data corroborate the claims made by Devaux et al., 2015 [10] and Ji et al., 2015 [24] that levels of Mir-133a are increased to a greater degree during STEMI as opposed to NSTEMI, and contradict the results reported by Li et al., 2013 [19] who reported no significant difference between the two. This difference in results may stem from the overall smaller sample size that was used or the relatively small number of NSTEMI patients included in the report by Li et al., 2013 [19].

We hypothesize that specific entities of myocardial injury may be characterized by distinct Mir-133a increase patterns. Yet, a concise meta-analysis of the literature yielded limited data in this regard. The included studies on Mir-133a did not specifically evaluate those with TO and STO in NSTEMI. Therefore, it is unknown whether NSTEMI patients with TO of culprit artery will present with similar Mir-133a concentrations as the STEMI group, but if such a correlation can be found, it may be used to identify these higher risk patients. Moreover, the underlying cause of myocardial damage in the included studies was not reported, and so the cases likely include different entities of NSTEMI such as myocardial infarction with nonobstructive coronary arteries (MINOCA), microvascular dysfunction, and vascular anatomies with varying degree of occlusion. It is unknown whether these entities may also present with markedly different Mir-133a elevation patterns, which might have diagnostic importance prior to coronary angiography. Additionally, since the present NSTEMI groups may contain a significant number of TO patients, it is possible that subanalysis of non-TO NSTEMI groups will demonstrate an even greater relative increase in Mir-133a plasma concentration relative to the TO group.

Further studies specifically designed to answer these questions are necessary in order to fully assess Mir-133a’s diagnostic potential in this respect. Future studies should also focus on the correlation between Mir-133a and other cardiac biomarkers in various populations of cardiac patients, as well as on the association with plaque vulnerability. As such, it remains to be determined whether differences in Mir-133a plasma concentration and elevation dynamics in NSTEMI may be used to identify different underlying pathophysiologies, and whether these differences may be used to accurately stratify risk groups.

### 4.4. Study Limitations and Methodological Issues

During the systematic literature review we encountered several methodological issues that hindered our ability to perform full comparisons between the data in each paper. It is our contention that these differences in methods and designs are the primary cause of conflicting reports as to Mir-133’s diagnostic ability. A prime example of this is the “endogenous control” used for the qRT-PCR analysis of Mir-133a plasma concentration. Multiple studies used truly endogenous microRNAs such as Mir-16, Mir-17, and U6 [11,18,24,27,29], though these controls, and especially U6, have been found to vary markedly in the same patients [42,43] and therefore may be unsuitable as reference microRNAs. Other studies used arbitrary CT values or the median CT for comparison [21,36], and multiple studies used single or multiple *C. elegans* microRNA “spike ins” [10,12,17,19]. To further complicate the picture, no uniform method of fold-change calculation was used. This resulted in non-standardized data, which makes for a major limitation when attempting to perform comparisons or draw conclusions from a meta-analysis. Furthermore, no standard protocol, equipment, or probes (SYBR/TaqMan) were implemented. In this study we relied primarily on AUC values to avoid these limitations, but in the case of distinguishing STEMI from NSTEMI only fold change data was available, so our conclusions in that instance may be limited.

Another significant design issue is the selection of controls. As we showed above, the pooled AUC value for Mir-133a was significantly greater in studies that used healthy controls as opposed to unhealthy controls. This fact limits analyses conducted by combining these two groups, and likely explains, at least in part, the large variations reported in the Mir-133a’s diagnostic ability.

Plasma concentration dynamics of Mir-133a in AMI are not yet fully understood. It is possible that time is a key factor in Mir-133a’s sensitivity and specificity. Due to limited reporting of precise times in the published literature, we were unable to conduct a thorough investigation of the impact that time has on the reported results. Though we showed that Mir-133a’s AUC is actually increased in studies with a longer duration from the onset of symptoms until sample acquisition, this conclusion is limited because the time windows are large, and the timing of individual measurements is not reported.

The studies included in this meta-analysis ranged in number of subjects from 9 in the smallest to 233 in the largest. To account for these differences the studies were weighted according to their size (n). While this is common practice, it does introduce a strong bias towards the larger studies, which also limits the conclusions drawn in this analysis.

We have posited the hypothesis that a contributing factor to the difference between Mir-133a plasma concentration in STEMI vs. NSTEMI is the degree of occlusion of the culprit artery, and therefore, that Mir-133a may be used for NSTEMI risk stratification. Since there are varied species of NSTEMI (MINOCA, TO, STO, etc.) that result from different underlying pathophysiologies that might each effect Mir-133a concentrations differently, further studies assessing the causal relation between degree of occlusion in NSTEMI and Mir-133a plasma concentration will be necessary to determine whether this hypothesis is true.

Finally, in order to rigorously test the clinical potential of Mir-133a in the setting of AMI, further studies will be needed with larger sample sizes, accurate timeline assessments, standardized methods of Mir-133a plasma concentration quantification, use of accepted reference values, and separate analyses based on subgroups.

## 5. Conclusions

Mir-133a has been investigated for its diagnostic potential for over a decade, yet a conclusive answer as to its clinical applicability is still lacking. In this meta-analysis we found that Mir-133a does possess a diagnostic ability (pooled AUC of 0.73), though it remains inferior to existing modalities [38,44]. Furthermore, we speculated Mir-133a may have an unrealized potential as a biomarker for the identification of high risk NSTEMI patients, and we suggest that it may be useful for detecting specific kinds of cardiac injuries and false-positive cardiac troponin increases. Further research will be needed in order to determine Mir-133a’s clinical applicability in these various scenarios. Lastly, we highlighted several significant methodological issues that prevent accurate comparisons between studies in this field and may be the cause of incongruent results.

## Figures and Tables

**Table 1:** Data summary from included papers.

Study (Author, Year, Reference)	Country	Number of Patients (Case/Control)	Patient Characteristics (Case)	Mean Age (Case)	Patient Characteristics (Control)	AUC	Mir Fold Increase (Total/STEMI/NSTEMI)	Max Time from Onset Until Sample Acquisition
Gidlof, O. et al. 2011 [21]	Sweden	9/11	STEMI Patients Undergoing PPCI	64.56 ± 2.7	STEMI/Healthy	0.859	70	12 h
Wang, G-K. et al. 2010 [22]	China	33/33	STEMI and NSTEMI	63.5 ± 10.1	AMI/Non-AMI ACS	0.867	____	12 h
Li, Y. et al. 2013 [19]	China	67/32	STEMI (*n* = 44) and NSTEMI (*n* = 23)	63.84 ± 11.17	AMI/Healthy	0.947	5.67	12 h
Devaux, Y. et al. 2015 [10]	Czechia, Italy, Poland, Spain, Switzerland	224/931	STEMI (*n* = 45) and NSTEMI (*n* = 179)	72	AMI/Non-AMI ACS	0.53	____	12 h
Wang, R. et al. 2011 [11]	China	58/21	STEMI and NSTEMI	60.06 ± 11.53	AMI/non-AMI ACS	0.89	4.4	24 h
Peng, L. et al. 2014 [18]	China	76/110	STEMI (*n* = 25) and NSTEMI (*n* = 51)	64.6	AMI/non-AMI ACS	0.912	7.26/7.6/7.1	____
Ji, Q. et al. 2015 [24]	China	98/23	STEMI (*n* = 77) and NSTEMI (*n* = 21)	62.33 ± 13.9	AMI/Healthy	0.787	15.26/16.65/10.9	24 h
Jia, K.-G. et al. 2016 [17]	China	233/146	STEMI (*n* = 156) and NSTEMI (*n* = 77)	62.32	AMI/Healthy and Non-AMI ACS	0.667	5.99/6.39/5.18	12 h
Liu, G. et al. 2018 [20]	China	145/30	NSTEMI Patients	67	NSTEMI/Healthy	0.927	2.4	12 h

AMI—acute myocardial infarction; STEMI—ST elevation myocardial infarction; NSTEMI—non-ST elevation myocardial infarction; AUC—area under the curve.

## Supplementary Materials

The following are available online at https://www.mdpi.com/2073-4409/9/4/793/s1, Figure S1: Trends as a function of %males, Figure S2: Trends as a function of age, Tables S1–S3: Complete Search Strategy. Table S4: PRISMA guidelines checklist.
