# Supplementary material for: The Diagnostic Value of Mir-133a in ST Elevation and Non-ST Elevation Myocardial Infarction: A Meta-Analysis
Source: Cells. 2020 Mar 25;9(4):793. doi: 10.3390/cells9040793 (PMC7226415; doi:10.3390/cells9040793)
Supplement: Supplementary file 1 [file cells-09-00793-s001.zip › cells-09-00793-s001 edited/Supplementary Figures S1-2 revised.docx]

| 2  4  6  8  10  12  14  16  0  20  40  60  80  100  Males (%)  Mir133a Increase (fold)  *n* = 601  r = 0.59; *p* = 0.219  (**a**) | 0.5  0.6  0.7  0.8  0.9  1.0  0  20  40  60  80  100  Males (%)  AUC  *n* = 858  r = 0.56; *p* = 0.193  (**b**) |
| --- | --- |

**Figure S1.** Linear regression analysis of (a) Relative increase (in fold) of Mir-133a plotted as a function of percentage of patients that are male. r =0.59. (b) AUC of Mir-133a plotted as a function of percentage of patients that are male. r = 0.56.

| 0  10  20  30  40  50  60  70  60  61  62  63  64  65  66  67  Age (years)  Mir133a Increase (fold)  *n* = 677  r = 0.16; *p* = 0.730  (**a**) | 0.5  0.6  0.7  0.8  0.9  1.0  60  62  64  66  68  70  72  Age (years)  AUC  *n* = 858  r = 0.54; *p* = 0.170  (**b**) |
| --- | --- |

**Figure S2.** Linear regression analysis of (a) Relative increase (in fold) of Mir-133a plotted as a function of mean age of participants, r=0.16. (b) AUC of Mir-133a plotted as a function of mean age of participants, r = 0.54
